# Associated factors of the co-occurrence of trachoma and soil-transmitted helminthiases in children 1 to 9 years old in rural communities of the Amazon basin in Loreto Department, Peru: Results from a population-based survey

**DOI:** 10.1371/journal.pntd.0010532

**Published:** 2022-07-25

**Authors:** Martha Idalí Saboyá-Díaz, Cristiam Armando Carey Angeles, Rosario del Socorro Avellaneda Yajahuanca, Salvith Karen Meléndez Ruíz, Rufino Cabrera, Harvy Alberto Honorio Morales, Paul E. Pachas, Monica Guardo, Kristen K. Renneker, Beatriz E. Muñoz, Sheila K. West

**Affiliations:** 1 Communicable Diseases and Environmental Determinants of Health Department, Pan American Health Organization, Washington, District of Columbia, United States of America; 2 Epidemiology Directorate, Regional Health Directorate of Loreto, Iquitos, Loreto, Peru; 3 Independent consultant, Lima, Peru; 4 Eye Health and Blindness Prevention Strategy, Regional Health Directorate of Loreto, Iquitos, Loreto, Peru; 5 General Directorate of Strategic Interventions in Public Health, Ministry of Health of Peru, Lima, Peru; 6 National Center of Public Health, National Institute of Health of Peru, Lima, Peru; 7 Health Surveillance, Disease Prevention, and Control, Pan American Health Organization, Guatemala City, Guatemala; 8 International Trachoma Initiative, The Task Force for Global Health, Decatur, Georgia, United States of America; 9 Dana Center for Preventive Ophthalmology, Wilmer Eye Institute, Johns Hopkins University, Baltimore, Maryland, United States of America; International Atomic Energy Agency, AUSTRIA

## Abstract

**Background:**

There is evidence of the occurrence of trachoma in Peru, and studies have shown that soil-transmitted helminthiases (STH) are affecting rural communities in the Amazon basin in Loreto Department. This study was done to estimate trachoma prevalence, STH prevalence, and the associated factors for both diseases in children aged 1–9 years in rural communities of Peru.

**Methodology:**

A population-based cross-sectional survey was carried out in rural communities of Loreto. A standardized survey questionnaire with individual and household risk factors related to both diseases was used. Ocular examination was done for all participants aged one year and above, and eye swab samples were collected from children with follicular trachoma (TF). Anthropometric measurements, stool samples for STH, and blood samples for hemoglobin measurement were taken from children.

**Principal findings:**

TF prevalence was 7.74% (95% CI 5.08–11.63%), STH prevalence was 49.49% (95% CI 25.00–52.43%), and prevalence of co-occurrence of both diseases was 5.06% (95% CI 2.80–8.98%) in children aged 1–9 years. Being at age 3–8 years old (AOR = 6.76; 95% CI 1.346–33.947), have an unclean face (AOR = 24.64; 95% CI 6.787–89.444), and having been dewormed in the last six months (AOR = 2.47; 95% CI 1.106–5.514), were risk factors of TF. Being a female (AOR = 0.22; 95% CI 0.103–0.457) was associated with decreased odds of TF. Having been dewormed in the last six months (AOR = 0.30; 95% CI 0.139–0.628) was a preventative factor for STH. Risk factors for children with both diseases mirrored the findings for risk factors for individual diseases.

**Conclusions:**

Neglected tropical diseases and associated risk factors overlap in communities living in vulnerable conditions in the Amazon basin of Peru. These findings support the need to implement integrated interventions, including mass drug administration, water, sanitation, and hygiene for both diseases in the study area.

## Author summary

Trachoma is an infectious disease caused by the bacterium *Chlamydia trachomatis*, transmitted through person-to-person contact, and it is the world’s leading infectious cause of blindness. Soil-transmitted helminths (STH) is a group of intestinal parasites composed of *Ascaris lumbricoides* (roundworms), *Trichuris trichiura* (whipworms), and *Ancylostoma duodenale* and *Necator Americanus* (hookworms), that are transmitted by fecal contamination of the soil and adversely affect nutritional status and impair cognitive processes in children. Although there is evidence of the occurrence of trachoma in several areas of Peru, an epidemiological study has not been carried out to determine if it is a public health problem. STH infections have been reported to affect children in areas of the Loreto Department in Peru, but prevalence surveys in rural communities bordering Brazil and Colombia have not been carried out. In this study, we present the results of an integrated survey for trachoma and STH in Loreto and the factors associated with the occurrence of these diseases. The results of this study show that both diseases and risk factors overlap, supporting the need to implement integrated interventions to eliminate them as public health problems in communities living in vulnerable conditions in the Amazon basin of Peru.

## Introduction

Trachoma is the leading infectious cause of blindness worldwide. It is caused by infection with *Chlamydia trachomatis* and is characterized by inflammatory changes in the conjunctiva in children with subsequent scarring, corneal opacity, and blindness in adults [1]. Active trachoma, characterized by the presence of subepithelial follicles in the upper tarsal conjunctiva (Trachomatous inflammation-Follicular (TF) and/or Trachomatous inflammation-Intense (TI)), is usually seen in childhood. Repeated and prolonged episodes of infection and inflammation can result in the scarring complication of trachoma, seen in late childhood and adults. Contraction of the scar tissue can cause in-turning of the eyelids (entropion), which can lead to the eyelashes touching the eyeball (Trachomatous trichiasis). Eventually, this can cause corneal opacification and blindness [2]. Trachoma is a public health problem in at least part of 44 countries in which it was estimated that 154.5 million people lived in areas that warranted interventions in 2020 [3]. Approximately, 1.8 million people were affected by trichiasis in 2020 [4,5]. The World Health Organization (WHO) promotes an integrated package of interventions (SAFE strategy) to eliminate trachoma as a public health problem. SAFE stands for surgery to correct trichiasis, antibiotics to reduce community infection, facial hygiene to reduce transmission in children, and environmental improvement actions to maintain low transmission [6].

Until 2016, trachoma was known as a public health problem only in three countries in Latin America (Brazil, Colombia, and Guatemala) while Peru and Venezuela were classified by WHO as countries with trachoma status uncertain [7]. Mexico was granted validation of the elimination in 2017 [8]. Since there were historical reports of the occurrence of trachoma cases in several areas in Peru [9], but not prevalence data to know if it was a public health problem, and there are communities of the country living in border areas with Brazil and Colombia where trachoma is endemic [10], it was considered necessary to carry out a population-based survey of trachoma in rural communities of the Amazon basin in the Department of Loreto.

Another neglected tropical disease associated with poverty is Soil-transmitted helminthiases (STH). It is caused by a group of intestinal nematodes composed of *Ascaris lumbricoides* (roundworms), *Trichuris trichiura* (whipworms), and *Ancylostoma duodenale* and *Necator Americanus* (hookworms). Infections caused by soil-transmitted helminths are transmitted by fecal contamination of the soil and adversely affect nutritional status and impair cognitive processes in children [11]. It was estimated that 295.5 million preschool children and 739.4 million school-age children required deworming for STH in 93 countries in 2020 [12]. WHO recommends periodical deworming to eliminate infecting worms, health education to prevent re-infection, and improved sanitation to reduce soil contamination with infective eggs [13,14].

In Latin America, 57,9 million children aged 1–14 years needed deworming for STH in 20 countries in 2020 [12]. Studies carried out to evaluate the impact of a deworming program in districts of the Department of Loreto in Peru, showed basal STH prevalence in preschool and school-age children between 14.5% and 72.2% [15–19]. Important reductions in the prevalence of STH were shown after deworming (e.g., 69.4% basal prevalence of *A*. *lumbricoides* that dropped to 1.8%). However, rural communities bordering Colombia and Brazil in the Loreto Department, that have insufficient access to safe water and sanitation, and not optimal deworming coverage [20], have not been evaluated to determine the epidemiological status of STH. Trachoma and STH are diseases affecting poor, unprivileged, and socioeconomically disadvantaged communities [21,22], which are characteristics of the populations living in the Amazon basin in the border of Peru with Brazil and Colombia. This justified the need of carrying out an integrated population-based survey to use resources efficiently to collect information to support the decision-making on implementing integrated public health interventions in the most in need population groups.

The purpose of the study was to determine the prevalence of active trachoma in children aged 1–9 years, trichiasis in population aged 15 years and older, prevalence and intensity of infection of STH in children aged 1–9 years, and the associated factors for the two diseases in rural communities of the Department of Loreto in Peru bordering Brazil and Colombia.

## Methods

### Ethics statement

The protocol of the study was approved before starting the survey by the ethics review committees of the National Institute of Health of Peru (resolution RD 0115-2017-OGITT-OPE/INS) and the Pan American Health Organization (reference PAHO-2017-03-0026). Written informed consent was obtained from the participating adults as well as from parents and guardians of all children aged 1–17 years; also, written assent was obtained from children aged 7–17 years. All information collected was stored and managed confidentially. Meetings with representatives of the indigenous organizations of the area of the study and with national and local authorities were held several months before starting the study to inform, coordinate, and obtain its approval.

### Study area

Loreto is the largest department of Peru with 368,851 square kilometers (28.7% of the national territory) located in the Amazon basin. It has 3,891 km of international borders with three countries: to the North-west with Ecuador (1,285 km), to the North-east with Colombia (1,515 km), and to the East with Brazil (1,154.3 km) being 38% of the international borders of the country [23]. The movement of people in this triple border is important from the socioeconomic and cultural perspective [23]. It is divided into eight provinces with a total population of 1,039,372 inhabitants (Census data of 2015) out of which 454,972 were younger than 15 years old, and most of the population lived in the urban areas (67.31%) [24,25]. The population density was 2.8 people per square kilometer in 2015 with approximately 10% of indigenous populations [25]. According to some studies, 56% of its population lives in poverty and 27.3% in extreme poverty [26]. Its altitude ranges between 61 and 220 meters above sea level, and its temperature varies between 22 and 32 Celsius degrees throughout the year. The relative humidity is 84%, and precipitations ranged between 2,000 and 3,000 millimeters per year [27].

Three provinces of Loreto bordering Brazil and Colombia (Mariscal Ramon Castilla, Putumayo, and Requena) were chosen to carry out the survey. These provinces were considered likely at the highest risk of trachoma based on historical reports of trachoma cases published in a study that compiled data between 1895 and 2000 [9] and because they share borders with trachoma foci in Brazil and Colombia [10]. These provinces were also considered at high risk of STH infections due to previous studies showing high prevalence and intensity of infections in preschool and school-age children in rural communities of Iquitos, capital of Loreto’s Department [15,17], reported low deworming coverage [20], and insufficient access to safe water and sanitation [23]. The latter are also known risk factors associated with the occurrence of trachoma. These three provinces shared similar demographic, ecological, socioeconomic, and climate conditions. Geographical access to these provinces is particularly difficult because roads are almost inexistent in the Department [27]. The communities can be reached mostly by boat, and in some areas only after several days.

### Study design and sample size

A population-based cross-sectional survey was carried out in rural communities of the Department of Loreto, Peru between May and July 2017 following the recommended standardized methodology of WHO in which trachoma prevalence should be estimated at the district level, with population sizes between 100,000 and 250,000, as this is the typical administrative unit for implementation of SAFE strategy [28]. Three provinces were selected to form the district for the study in Loreto (Mariscal Ramon Castilla, Putumayo, and Requena) with a total population of 158,807. The sample size for the study was calculated using the single population proportion for precision formula [29], with an expected 10% prevalence of TF in children 1–9 years old, an absolute precision of ±3%, a design effect of 2.65 (based on recommendations from other studies) [30], and a non-response factor of 1.2. As a result, we estimated a total of 1,222 children aged 1–9 years to be sampled. The power of the sample size with an expected 10% prevalence of TF was considered adequate to estimate also an expected 20% prevalence of STH with an absolute precision of ±5%.

### Sampling selection

#### Selection of clusters

A two-stage cluster random sampling strategy was carried out. In the first stage, 20 clusters (communities) were randomly selected, with a probability of selection proportional to the size, out of 113 rural communities (150 to 500 inhabitants per community) located in four river basins identified in the three provinces of the district of evaluation. Urban areas were excluded from the sampling frame because they are considered at low risk of trachoma and STH. Communities with less than 150 inhabitants were grouped with geographically close communities to represent a single cluster while communities with more than 500 inhabitants were divided into segments to represent individual clusters for the sampling frame. Communities with safety issues or so isolated that reaching them was extremely expensive were excluded from the sampling frame.

#### Selection of households and participants

In the second stage, 30 households were randomly selected in each cluster. To avoid convenience sampling of children, a fixed number of households to be enrolled per cluster was determined, based on the number of households (generally ≤30) that a team should be able to complete in a day [30]. Upon arrival at each cluster, the field teams drew maps of the distribution of the households to randomly select the 30 households for the study. If the community had 30 households or less, all the households were visited; if the community had 31–60 households, 30 households were selected randomly using a table of random numbers; if the community had 61 or more households, the community was divided into segments of similar size allowing each segment to have at least 30 households, then a segment was selected randomly, and all households were visited. In each household selected, all participants aged 1 year and above that consented to participate were examined for trachoma, and children aged 1–9 years were examined for STH.

### Household interviews

After consenting to participate in the study, the household’s GPS coordinates were collected and an interview with the head of each household selected was completed to collect data on demographic characteristics of all residents as well as on drinking and face washing water source, distance to bring drinking and washing faces water, place to defecate, and place to dispose feces of children under five years old. The head of each household was also interviewed to collect data on hygiene behaviors of children 1 to 9 years related to STH (frequency of playing with earth, washing hands before eating and after defecating, wearing footwear that protects from contact with earth, and history of deworming in the last six months). By inspecting the household, data were collected on the type of latrine, facility to washing hands close to the toilet or latrine, water and soap available at the facility to washing hands, type of floor of the house, and the number of rooms to sleep. These interviews were carried out by trained recorders using a standardized questionnaire developed for trachoma surveys by Tropical Data [30] into which some water, sanitation, and hygiene questions related to STH were included.

All consenting/assenting residents aged 1 year and above were assessed for trachoma signs using the WHO simplified grading system, and children aged 1 to 9 years were assessed for facial cleanliness by recording the presence of eye secretion, nasal discharge, and flies on the face. Swab samples of the upper tarsal conjunctiva were collected from children aged 1 to 9 years with active trachoma signs for the identification of *C*. *trachomatis*. All consenting/assenting children aged 1 to 9 years were assessed for hemoglobin levels and STH infections by collecting and analyzing capillary blood and stool samples, respectively; data of height and weight were also recorded for participants in this age group. To increase the screening rate, the survey teams revisited at least once the households where censused individuals were absent by the time of the first visit. If after the second visit the participant was not found, it was recorded as absent.

### Survey teams

Survey teams comprised a trachoma grader, a data recorder, a laboratory technician, and a riverboat driver. Trachoma graders were trained by certified trainers of Tropical Data using the training manual version three which is based on the WHO simplified grading system [31]. Data recorders were trained by a certified Tropical Data recorders trainer to use a smartphone device to enter data on trachoma examination for all eligible participants as well as on data from the household interviews [31]. Laboratory technicians were trained by a senior laboratory trainer on the Kato-Katz technique using the WHO manual for the diagnosis of intestinal parasites [32] and on the determination of hemoglobin using a portable hemoglobinometer [33]. Laboratory technicians were also trained on anthropometric measurements using the national guidelines of growth and development for children [34].

Survey teams were trained for one week on the procedures of the survey protocol and a supervisor accompanied the survey teams at least once for one week to review the progress on the fieldwork, the implementation of the procedures of the survey, and to provide recommendations according to the supervision results.

### Field and laboratory procedures

For trachoma examination, both eyes of each eligible participant were examined using a magnification 2.5x binocular loupe and a torch. Trachoma graders cleaned their hands with antibacterial gel between each examination to reduce the risk of contamination. Every child with TF and/or TI had a swab taken of the upper right conjunctiva for detection of *C*. *trachomatis*. Eye swab samples of two children who did not have trachoma were taken as a negative control in each cluster of the study. Also, two swab samples were taken in each cluster to monitor field or laboratory contamination by waving a swab in the air over a child. The swabs were kept and transported cold in the field (2–8°C) and frozen (-20°C) when returned to the laboratory at the National Institute of Health of Peru. All the specimens were shipped on dry ice to the International Chlamydia Laboratory at Johns Hopkins Medical Institutions where detection of *C*. *trachomatis* DNA was performed using the APTIMA Combo 2 Assay test (Hologic, Inc. Partner System, San Diego, CA, USA), according to manufacturer instructions [35,36]. Children with TF or TI and their household members received a dose of the antibiotic recommended by WHO and the International Trachoma Initiative according to age and height [6,37]. Contact information of TT cases was recorded to locate them later for ophthalmologic evaluation and eventual treatment.

All eligible children aged 1–9 years were asked to provide one stool sample early in the morning of the survey day for which a container was provided to the parents and guardians the day before with instructions for collecting the sample. Stool samples were analyzed by the laboratory technician as soon as they were collected in each cluster using the Kato-Katz technique [32] for identification and counting of eggs of STH. The intensity of infection was analyzed as eggs per gram of stool and classified according to WHO guidelines (Table 1) [14]. Ten percent of stool samples were randomly selected and preserved in formol 10% to evaluate the concordance on the diagnosis of STH. All participant children aged 2–9 years received a dose of albendazole (400 mg) at the time of the survey as recommended by WHO for STH preventive chemotherapy [13]. There was no coadministration of antibiotics and anthelmintic medicines.

**Table 1:** Classification criteria for the intensity of infection of soil-transmitted helminths.

STH infection	Intensity of infection (eggs per gram)		
	Mild	Moderate	Severe
*Ascaris lumbricoides*	1–4,999	5,000–49,999	≥50,000
*Trichuris trichiura*	1–999	1,000–9,999	≥10,000
Hookworms	1–1,999	2,000–3,999	≥4,000

The weight of each eligible child aged 1–9 years was measured using a digital scale after removing shoes and heavy clothes. Height was measured using a vertical height scale after removing shoes and head ornaments, and making the child stand with feet flat, together, and against the flat surface of the scale. Measurement of weight and height followed the Ministry of Health of Peru guidelines for anthropometric measurements in children [34].

A blood sample from a finger prick was taken for evaluation of hemoglobin levels from each eligible child aged 1–9 years. Blood samples were analyzed for anemia using a portable hemoglobinometer. Values below 11 mg/dl and 7.0 mg/dl for children aged 1–4 years and below 11.5 mg/dl and 8.0 mg/dl for children aged 5–9 years were classified as anemia and severe anemia, respectively, based on the WHO recommendations [38].

### Data collection, management, and analysis

All data were captured electronically in smartphones using a customized LINKS application developed by the Global Trachoma Mapping Project (GTMP) (Task Force Links/Task Force for Global Health, Decatur, GA, USA) [30,39] and managed by Tropical Data [40]. Data collected in the field were transferred to a central cloud-based reporting and data management system. Data clean-up was done before the analysis following the Tropical Data procedures that are described elsewhere [30]. Data were transferred to Stata 13.1 for analysis.

Analysis of data on prevalence and risk factors was done using sample weights for the two-stage cluster sampling, and by using statistical analysis for complex sampling procedures. Descriptive analysis such as percentages and means were used for demographic, trachoma, STH, anemia, weight, and height data. Bivariate analysis of the prevalence of trachoma and STH was done with socio-demographic indicators, children’s risk factors and behaviors, and household characteristics. A map with the spatial distribution of clusters surveyed was drawn using Tableau 10.5.3.

For the analysis of anthropometric measurements, WHO Child Growth Standards for children aged 0–5 years [41] and WHO Reference 2007 standards for 5 to 19 years old [42] were used for the classification of body mass index (BMI). Anthropometric data were analyzed using Anthro-plus [43].

Multinomial logistic regression models with cluster adjustment were used to identify factors associated with trachoma alone, STH alone, or both in children aged 1–9 years. Variables associated with the outcomes at a *p*-value less than 0.2 in the bivariate analysis were included in the multivariate phase. A backward stepwise elimination procedure was used to reach a parsimonious model (i.e., all factors included were independently associated at a significance level ≤0.05).

## Results

### Population surveyed

**Fig 1:**
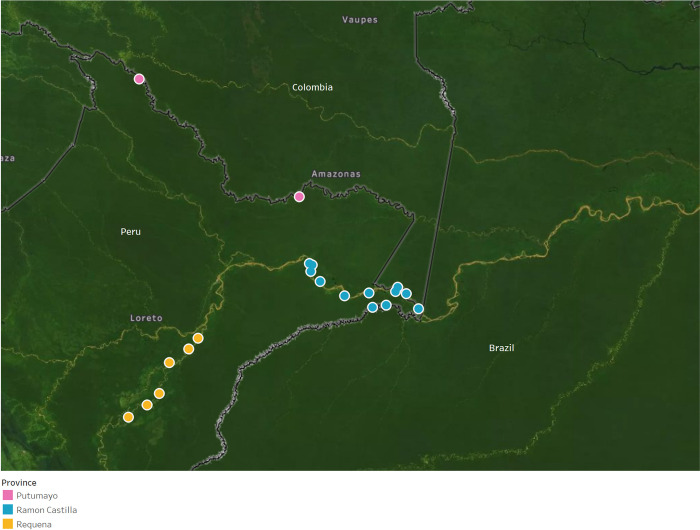
Clusters included in the study area by province in rural communities of Loreto Department, Peru 2017. (Figure created using Tableau Desktop (Seattle, USA) using a base map licensed under the Open Data Commons Open Database License by the Open Street Map Foundation).

A total of 2,289 people were surveyed out of which 1,221 were children aged 1–9 years (52.59%) and 916 were 15 years and above (40.42%) in 20 clusters (Fig 1). Most of the respondents, 1,923 (82.79%), were mestizos (Self-identification of a person of being ethnically mixed between white and indigenous ancestry) and 366 indigenous (17.21%) (Table 2). There were 15 children 1–9 years old that after being visited twice were registered as absents and treated as non-responders.

**Table 2:** Characteristics of study participants and children aged 1–9 years examined in rural communities of Loreto Department, Peru, 2017.

Characteristic	N	(%)^a^
**Total surveyed population**	2289	
Sex		
Male	959	42.41
Female	1330	57.59
Age (years)		
1–9	1221	52.59
10–14	152	6.99
≥15	916	40.42
Ethnicity		
Indigenous	366	17.21
Mestizo	1923	82.79
**Examined children (1–9 years) characteristics**	1221	
Sex		
Male	619	50.18
Female	602	49.82
Province		
Mariscal Ramon Castilla	754	63.61
Requena	342	23.58
Putumayo	125	12.80

^a^Percentages weighted.

### Trachoma clinical signs

Out of 1,221 children aged 1–9 years who were examined for trachoma, 97 had TF out of which 61 (63.91%) were male, and 79 (81.81%) were children aged 3–8 years. Ninety-four (97.09%) TF cases were in mestizos. The prevalence of TF adjusted by sampling strategy was 7.74% (95% CI 5.08–11.63%). The intra-cluster correlation coefficient for TF was 0.24 (95% CI 0.06–0.43) which shows that children with TF clustered within the community. A total of 97 eye swab samples of children with TF, 25 eye swab samples of children without TF, and 25 contamination control swab samples were collected. None of the TF cases or control samples had a positive laboratory test for *C*. *trachomatis*. A total of five cases of trichiasis, out of which three were TT cases (includes scarring), were identified out of 916 people aged ≥15 years examined. The adjusted prevalence of TT by age and sex was 0.13%. (95% CI 0.00–0.32%). The three TT cases were in mestizos from Requena province, and 2 out of the 3 cases were in females.

### Soil-transmitted helminthiases

Out of 1,110 children aged 1–9 years who were examined for STH, 536 (49.49%; 95% CI 25.00–52.43%) were infected with any STH. *Ascaris lumbricoides* was the most prevalent species found in 406 children (37.16%; 95% CI 29.90–45.28%) followed by *Trichuris trichiura* (22.84%; 95% CI 14.90–33.35%), and hookworms (1.78%; 95% CI 0.73–4.30%). The prevalence of any STH was higher in females (51.19%; 95% CI 24.99%-54.13%). Prevalence of ascariasis and trichuriasis were higher in females (39.57% and 23.10%, respectively) while the prevalence of hookworms was higher in males (2.51%) (Table 3). Data on the intensity of infection was available for 530 out of the 536 children infected with any STH. Most children had mild infections (73.10%), and one in five had moderate infections (24.78%) (Table 4). The intra-cluster correlation coefficient for any STH was 0.21 (95% CI 0.08–0.34) which shows that children who had STH clustered within the community. The prevalence of infection with any STH was estimated based on the method described by Silva and Hall [44]. The ninety-eight stool samples collected for analysis of diagnosis concordance showed that concordance on positive samples for *T*. *trichiura* was 37.14%, hookworm 50%, and *A*. *lumbricoides* 82.22%. It was reported that some samples (no exact number reported) were highly diluted in formol causing problems in the microscopic reading, and low sensitivity (below 50%) of the laboratory test used for STH species (spontaneous sedimentation) [45]. Hence, concordance results were not conclusive.

**Table 3:** Children surveyed and infected with soil-transmitted helminths according to demographic characteristics, rural communities of Loreto Department, Peru 2017.

Characteristic	Children surveyed	*Ascaris lumbricoides*		*Trichuris trichiura*		Hookworms		Any STH		
		n	%^a^	n	%^a^	n	%^a^	n	%^a^^,^^b^	(CI 95%)
Age (years)										
1–2	226	92	41.31	39	17.67	4	1.71	105	49.61	25.00–52.55
3–8	753	269	36.05	167	23.66	11	1.63	366	49.37	25.00–52.31
9	131	45	36.50	32	26.71	3	2.66	65	52.25	24.95–55.10
Sex										
Male	574	197	34.90	124	22.60	13	2.51	272	48.54	24.98–51.48
Female	536	209	39.57	114	23.10	5	0.99	264	51.19	24.99–54.13
Total	1110	406	37.16	238	22.84	18	1.78	536	49.49	25.00–52.43

^a^Percentages weighted.

^b^Adjusted by Silva and Hall method: P_*ath*_ = (a+t+h)—(a*t + a*h + t*h) + (a*t*h) / 1.06; where a = prevalence of Ascariasis (expressed as a proportion); t = prevalence of Trichuriasis (expressed as a proportion); h = prevalence of hookworm infection (expressed as a proportion).

**Table 4:** Children surveyed and infected by the level of intensity of infection of soil-transmitted helminths, rural communities of Loreto Department, Peru 2017.

Type of STH infection	Children surveyed	Mild		Moderate		Severe	
		n	%^a^	n	%^a^	n	%^a^
Ascariasis	404	318	77.24	78	20.77	8	1.99
Trichuriasis	233	157	66.92	74	32.26	2	0.81
Hookworms	18	15	83.20	2	9.88	1	6.92
**Any STH** ^**b**^	**530**	**394**	**73.10**	**125**	**24.78**	**11**	**2.12**

^a^Percentages weighted.

^b^Intensity of infection with any STH: *Numerator*: number of children infected in each level of intensity with any of the three species of STH. *Denominator*: number of children infected with any of the three species of STH for which intensity data was available (n = 530).

### Anemia and body max index

The mean of hemoglobin in the 1,221 children 1–9 years old examined was 11.63 g/dl (95% CI 11.51–11.76 g/dl). A total of 404 (32.25%) were identified with anemia. Of 1,204 children 1–9 years old with measures of height and weight, 932 (77.13%) had a normal BMI. Two hundred thirty-one (19.68%) had a BMI above normal and 41 (3.19%) below normal. One hundred ten (17.24%) children aged 1–5 years, were at risk of being overweight, and 14 (2.18%) were severely wasted. Eighty-six (16.18%) children aged 6–9 years had overweight (Table 5).

**Table 5:** Anemia and Body Mass Index of children aged 1–9 years examined in rural communities of Loreto Department, Peru, 2017.

Characteristic	N	(%)^a^^,^^b^
Anemia^c^	1,221	
Non-anemia	817	67.75
Anemia	404	32.25
Severe anemia	0	0.00
Body Mass Index (BMI)^d^	1204	
Normal	932	77.13
Below normal	41	3.19
Above normal	231	19.68
BMI in children aged 1–5 years	649	
Overweight or obese	22	3.56
Possible risk of overweight	110	17.24
Normal	485	74.61
Wasted	18	2.42
Severely wasted	14	2.18
BMI in children aged 6–9 years	555	
Obesity	13	2.25
Overweight	86	16.18
Normal	447	79.97
Thinness	4	0.76
Severe thinness	5	0.84

^a^Percentages weighted.

^b^Where data were missing, percentages are based on denominators for which data are available.

^c^Adjustments by altitude were not needed because the study area was below 200 meters above sea level. According to WHO recommendations.

^d^Seventeen children had values of height or weight that were not adequate to calculate BMI. It could be related to mistakes in the recording process.

### Individual-level factors for trachoma and STH

Of 1,221 children 1–9 years old, ninety-two (7.71%) children had an unclean face (presence of eye secretion or nasal discharge or flies on the face at the time of the examination). The more common sign was eye secretion which was reported in 67 children (5.44%).

Most children, 1,049 (82.42%), reported playing with earth out of which 757 (70.38%) reported playing many times a day. Most children were reported washing their hands before eating and after defecating, 1,128 (90.46%) and 1,111 (89.10%), respectively. Two hundred seventy (20.91%) children received deworming treatment in the last six months (Table 6).

**Table 6:** Individual-level factors for trachoma and soil-transmitted helminthiases of children aged 1–9 years examined in rural communities of Loreto Department, Peru, 2017.

Characteristic	N	(%)^a^^,^^b^
Unclean face^c^	92	7.71
Eye secretion	67	5.44
Nasal discharge	35	3.33
Flies on face	3	0.34
Play with earth^d^		
Never	172	17.58
Yes	1049	82.42
Frequency of playing with earth per day^d^		
Rarely	2	0.16
Few times	290	29.46
Many times	757	70.38
Wash hands before eating	1128	90.46
Wash hands after defecating	1111	89.10
Wearing footwear that protects from contact with earth	32	2.99
Dewormed in the last six months	270	20.91

^a^Percentages weighted.

^b^Where data were missing, percentages are based on denominators for which data are available.

^c^Presence of eye secretion or nasal discharge or flies on the face at the time of examination.

^d^Frequency of playing with earth reported by the household head.

### Household-level factors for trachoma and STH

Of 1,221 children examined for trachoma, 792 (64.48%) lived in households in which the main source of drinking water was surface water (river, dam, lake, canal) which also was the main water source for washing faces for 1,037 (81.70%) children. Seven hundred six (59.41%) children lived in households where members have to walk less than 30 minutes to the main source of drinking water, and 848 (65.91%) children lived in households where members also have to walk less than 30 minutes to the source of water used for washing faces. Two hundred fifty-nine (26,61%) children washed their faces at the water source. Seven hundred seventy-six (66.40%) children lived in households without latrine or any other facility to defecate, and 445 (33.60%) had access to a shared, public, or private latrine. Four hundred fifty-seven (50.73%) children lived in households where feces of children under five years old were left in the open. Sixty-six (14.69%) children lived in households with a facility to wash hands near the toilet or latrine. Out of these, 64 (95.99%) had water available, and 62 (93.12%) had soap available. Almost all children, 1089 (99.43%), lived in households that had wood floors, and 228 (19.63%) in households without separated rooms to sleep (Table 7).

**Table 7:** Household-level factors for trachoma and soil-transmitted helminthiases of children aged 1–9 years in rural communities of Loreto Department, Peru, 2017.

Characteristic	N	(%)^a^^,^^b^
Drinking water source		
Surface water^c^	792	64.48
Rainwater collection	372	30.99
Other sources	57	4.53
Water source to wash faces		
Surface water^c^	1037	81.70
Rainwater collection	131	14.00
Other sources	53	4.30
Time to main source of drinking water, minutes^d^		
Water source in yard	392	32.14
Less than 30 minutes	706	59.41
Between 30 minutes and 1 hour	123	8.46
Time to main source of water used for face-washing, minutes^d^		
All face washing done at the water source	259	26.61
Less than 30 minutes	848	65.91
Between 30 minutes and 1 hour	114	7.49
Place to defecate		
No structure; outside elsewhere	776	66.40
Shared, public, or private latrine	445	33.60
Place to dispose child under 5 years old feces—left in the open	457	50.73
Facility to washing hands near the toilet/latrine	66	14.69
Water available at the facility to washing hands^f^	64	95.99
Soap available at the facility to washing hands^f^	62	93.12
Type of floor—wood	1089	99.43
Number of rooms to sleep- No rooms	228	19.63

(n = 1221)

^a^Percentages weighted.

^b^Where data were missing, percentages are based on denominators for which data are available.

^c^River, dam, lake, canal.

^d^Time for round-trip estimated by household head.

^e^Facility to washing hands within 15 meters of the toilet/latrine; Distance observed by the interviewer at the time of the visit to the household.

^f^Total out of the number of households with a facility to washing hands near the toilet/latrine (n = 66).

### Co-occurrence of trachoma and STH

A total of 55 children aged 1–9 years were infected simultaneously with trachoma and any STH (5.06%; 95% CI 2.80–8.98%). Thirty were males (57.16%) and 44 were aged 3–8 years (80.99%). Fifty-four were mestizos (98.27%).

### Factors associated with trachoma and STH in children

Being at age 3 to 8 years old, being mestizo, having an unclean face, and playing many times with earth per day were independently associated with increased odds of TF. However, confidence intervals were wide for all except for the age group. Being female was independently associated with decreased odds of TF. Distance to the main source of drinking water was not independently associated with TF (Table 8).

**Table 8:** Bivariate logistic regression analysis of factors associated with trachomatous inflammation-follicular (TF), among children aged 1–9 years, rural communities of Loreto Department, Peru 2017.

Characteristic	Presence of TF		OR with 95% CI	
	No	Yes	Crude^b^	*p*-value
*Individual*				
Age group (years)				
1 to 2	238	12	1	
3 to 8	745	79	2.02 (1.086–3.744)	0.028
9	141	6	0.63 (0.252–1.551)	0.293
Sex				
Male	558	61	1	
Female	566	36	0.54 (0.380–0.776)	0.002
Ethnicity				
Indigenous	165	3	1	
Mestizo	959	94	6.54 (1.137–37.608)	0.037
Province				
Mariscal Ramon Castilla	699	55	1	
Requena	300	42	1.86 (0.837–4.130)	0.119
Putumayo	125	0		
Unclean Face				
No	1072	57	1	
Yes	52	40	13.74 (5.291–35.656)	0.000
Eat earth				
No	1108	92	1	
Yes	16	5	3.82 (0.803–18.120)	0.088
Play with earth (per day)				
No or rarely	173	1	1	
Few times	278	12	6.08 (0.843–43.842)	0.071
Many times	673	84	18.32 (2.511–133.653)	0.006
*Household*				
Time to main source of drinking water, minutes^a^				
Water source in yard	360	32	1	
Less than 30 minutes	647	59	0.89 (0.4512–1.763)	0.729
Between 30 minutes and 1 hour	117	6	0.54 (0.270–1.073)	0.076

(n = 1221)

*OR* odds ratio.

^a^Time for round-trip estimated by household head.

^b^Bivariate logistic regression accounting for clustering.

Being mestizo, playing with earth a few and many times per day, and washing faces with collected rainwater were independently associated with increased odds of STH. Living in Putumayo province and having been dewormed in the last six months, were independently associated with decreased odds of STH. Anemia and BMI were not independently associated with STH (Table 9).

**Table 9:** Bivariate logistic regression analysis of factors associated with soil-transmitted helminthiasis (STH), among children aged 1–9 years, rural communities of Loreto Department, Peru 2017.

Characteristic	Presence of STH		OR with 95% CI	
	No	Yes	Crude^d^	*p*-value
*Individual*				
Ethnicity				
Indigenous	131	36	1	
Mestizo	443	500	4.65 (2.396–9.032)	0.000
Province				
Mariscal Ramon Castilla	327	330	1	
Requena	146	185	1.15 (0.590–2.250)	0.662
Putumayo	101	21	0.18 (0.072–0.467)	0.001
Play with earth (per day)				
No or rarely	105	39	1	
Few times	117	120	2.84 (1.370–5.890)	0.007
Many times	352	377	3.24 (1.425–7.346)	0.007
Wash hands before eating				
No	56	21	1	
Yes	518	515	2.74 (0.978–7.694)	0.055
Wash hands after defecating^a^				
No	59	31	1	
Yes	514	505	2.06 (0.790–5.343)	0.131
Dewormed against intestinal parasites in the last 6 months				
No	418	467	1	
Yes	156	69	0.40 (0.169–0.969)	0.043
Anemia				
No anemia	375	363	1	
Mild	109	100	0.99 (0.663–1.493)	0.982
Moderate	90	73	0.91 (0.489–1.699)	0.758
Severe	0	0	-	
Body Mass Index (BMI)^a^				
Normal	430	418	1	
Below normal	24	14	0.54 (0.237–1.236)	0.136
Above normal	112	96	0.91 (0.560–1.492)	0.705
*Household*				
Water source for washing face				
Surface water^b^	525	464	1.00	
Rainwater collection	30	48	2.03 (1.143–3.599)	0.018
Other sources	19	24	1.44 (0.563–3.672)	0.424
Time to main source of water used for face-washing, minutes^c^				
All face washing done at the water source	114	66	1	
Less than 30 minutes	410	406	1.91 (0.750–4.842)	0.164
Between 30 minutes and 1 hour	50	64	2.32 (0.925–5.805)	0.071
Place to dispose child under 5 years old feces^a^				
Child use toilet or latrine	40	67	1	
Disposed elsewhere	136	167	1.29 (0.759–2.189)	0.326
Left in the open	219	205	1.60 (0.913–2.810)	0.095

(n = 1110)

*OR* odds ratio.

^a^Where data were missing, totals are based on denominators for which data are available.

^b^River, dam, lake, canal.

^c^Time for round-trip estimated by household head.

^d^Bivariate logistic regression accounting for clustering.

Variables independently associated with increased or decreased odds of TF or STH at a *p*-value less than 0.2 in the bivariate analysis were included in the multinomial logistic regression. The multivariate analysis comparing children aged 1–9 years with either TF alone, STH alone, and co-occurrence of both diseases to the group of children without disease, showed that being at age 3 to 8 years old (AOR = 6.76; 95% CI 1.346–33.947), have an unclean face (AOR = 24.64; 95% CI 6.787–89.444), and having been dewormed in the last six months (AOR = 2.47; 95% CI 1.106–5.514), were independent predictors of TF in children. Being a female (AOR = 0.22; 95% CI 0.103–0.457) was associated with decreased odds of TF (Table 10). Having been dewormed in the last six months (AOR = 0.30; 95% CI 0.139–0.628) was associated with decreased odds of STH (Table 10).

**Table 10:** Multinomial logistic regression analysis of factors associated with trachomatous inflammation-follicular (TF), and soil-transmitted helminthiasis (STH), among children aged 1–9 years, rural communities of Loreto Department, Peru 2017.

Characteristic	TF alone		STH alone		TF and STH co-occurrence	
	Adjusted OR with 95% CI^b^^,^^c^	*p*-value	Adjusted OR with 95% CI^b^^,^^c^	*p*-value	Adjusted OR with 95% CI^b^^,^^c^	*p*-value
*Individual*						
Age group (years)						
1 to 2	1		1		1	
3 to 8	6.76 (1.346–33.947)	0.023	1.29 (0.948–1.763)	0.099	4.30 (1.704–10.858)	0.004
9	2.92 (0.389–21.991)	0.278	1.62 (0.960–2.738)	0.068	1.32 (0.404–4.306)	0.628
Sex						
Male	1		1		1	
Female	0.22 (0.103–0.457)	0.000	0.88 (0.656–1.193)	0.400	0.60 (0.384–0.945)	0.030
Province						
Mariscal Ramon Castilla	1		1		1	
Requena	0.75 (0.277–2.045)	0.557	1.28 (0.642–2.561)	0.458	4.30 (1.592–11.610)	0.007
Unclean Face						
No	1		1		1	
Yes	24.64 (6.787–89.444)	0.000	1.47 (0.688–3.129)	0.301	38.86 (14.290–105.660)	0.000
Dewormed against intestinal parasites in the last 6 months						
No	1		1		1	
Yes	2.47 (1.106–5.514)	0.030	0.30 (0.139–0.628)	0.003	0.39 (0.119–1.247)	0.105
*Household*						
Water source for washing face						
Surface water or other sources^a^	1		1		1	
Rainwater collection	1.18 (0.406–3.432)	0.747	1.30 (0.671–2.520)	0.413	6.74 (2.659–17.067)	0.000

(TF n = 35; STH n = 481; TF and STH n = 55)

*AOR* adjusted odds ratio.

^a^River, dam, lake, canal.

^b^Bivariate logistic regression accounting for clustering.

^c^Compared to the group without disease (n = 539).

The independent predictors of co-occurrence in children aged 1–9 years were being at age group 3–8 years old (AOR = 4.30; 95% CI 1.704–10.858), living in Requena province (AOR = 4.30; 95% CI 1.592–11.610), having an unclean face (AOR = 38.86; 95% CI 14.290–105.660), and washing faces with collected rainwater (AOR = 6.74; 95% CI 2.659–17.067). Being a female (AOR = 0.60; 95% CI 0.384–0.945) was associated with decreased odds of co-occurrence of TF and STH (Table 10).

Although ethnicity was associated with TF and STH in the bivariate analysis, after adjusting the multivariate model by the province of residence of the children, ethnicity was no longer a predictor for any of the two diseases or the co-occurrence of TF and STH.

## Discussion

The TF prevalence in children aged 1–9 years found in the rural communities of Loreto Department (7.74%) is similar to the prevalence reported in districts of Guatemala in 2011 [46] but lower than the reported in several districts of the Amazon basin in Colombia (TF prevalence between 10.00% and <30%) [47]. Although we were underpowered to report differences by province, it is interesting that no TF and TT cases were found in the Putumayo province where most of the population surveyed was indigenous. This province borders an Amazon district in Colombia that is known to have trachoma. This might be related to the small number of children examined in only two clusters. We had to join several communities in this province to form clusters to be part of the sampling frame. Another important aspect is that indigenous communities can have different living conditions and hygiene behaviors that might be related to risk factors of communicable diseases, including trachoma, depending on the ethnic group [48]. A more in-depth and powered study would be needed to confirm the findings. Because the entire district should be targeted with SAFE strategy interventions (based on the TF prevalence found in this study), it would be ideal to examine children and adults during the visits to the communities of Putumayo province as an opportunity to collect more information on the occurrence of TF cases, and TT cases to refer them to evaluation and surgery.

Infection with *C*. *trachomatis* was found negative in children aged 1–9 years with TF. Freezes and thaws might have affected the recovery of genetic material during the transport within and outside the country as reported in some other studies [49]. Another possible reason is that the follicles identified were not due to trachoma. Follicles are not pathognomonic of trachoma, and the differential diagnosis of follicular conjunctivitis includes, among others, other bacterial infections, and toxic conjunctivitis secondary to topical drugs or eye cosmetics [49,50]. Some photos of TF cases were shared with experts on trachoma in Colombia and at WHO, and they corroborated that the images were compatible with trachoma.

It is a challenge for the Peruvian trachoma program to have found no infection in a district with a prevalence of TF around 7% in children aged 1–9 years and 0.13% of TT in people aged 15 years and above. Although this finding might not be rare because a low prevalence of conjunctival infection with *C*. *trachomatis* in a treatment-naïve trachoma-endemic region was reported in the Solomon Islands [51], additional studies might be needed to better characterize this finding in the Amazon basin of Peru. There have been discussions about the interventions to implement when clinical signs of trachoma are present in children, but evidence of TT in adults, infection, and transmission markers does not seem to be correlated [52]. At this point, based on the prevalence of TF in the studied provinces of Loreto, considering that the studied population is living in bordering areas with trachoma foci in Brazil and Colombia, there are historical reports of trachoma in the country and these populations are living in vulnerable conditions, it is felt that the SAFE strategy should be implemented in the studied area following the current WHO recommendations.

Almost half of the children aged 1–9 years in the district surveyed had an infection with at least one species of STH (49.49%), despite deworming of children in Loreto Department since 2012 [20]. This might be related to the low coverage of deworming reported in our study (20.91%), but it could also be possible that parents and guardians did not know or remember if their children were dewormed at the schools, resulting in a memory bias. The deworming program in Loreto targets children aged 3–17 years through the school’s system [15], a strategy that might be left some unenrolled children without treatment [20]. There are no reports available on deworming coverage evaluation from rural and remote communities in Loreto or records to know if they are giving directly observed treatment to children in the study area. Non-treated at-risk groups (e.g., preschool-age children, childbearing women, etc.) in Loreto Department can contribute to contaminating the environment that in combination with lack of access to safe water and basic sanitation, keep the conditions for infection and reinfection in the communities, a finding reported in other studies in Loreto Department [16,20]. An in-depth analysis of STH deworming coverage would be needed to characterize better this finding.

The species more prevalent were *A*. *lumbricoides* and *T*. *trichiura* which is a common finding in other studies in Iquitos [16,19]. However, the prevalence of hookworm was very low. It might be possible that delays in the process to read the stool samples are related to the low prevalence of hookworm reported. Stool samples should be analyzed within 3 hours of the collection because hookworm eggs degenerate very soon [53]. The time delay between stool sample collection and processing, and between processing and microscopic reading was not recorded in our study. Kato-Katz technique is widely used for diagnosis of STH in epidemiologic surveys and recommended by the WHO for field diagnosis of STH due to its low cost, non-invasive nature, and the relatively low level of technical skill required [14,32]. Good sensitivity of a single stool examination using the Kato-Katz technique for detection of *A*. *lumbricoides* and *T*. *trichiura* has been reported, but relatively poor sensitivity for detection of hookworm infections [54]. However, some studies have reported low sensitivity, particularly for the detection of light-intensity helminth infections. This low sensitivity might cause underestimation of hookworm prevalence mainly when only one sample of stool is collected [55].

Although most of the infections with any STH were mild, 26.9% were moderate or severe infections, which can cause important morbidity in children [13,56,57]. High STH prevalence and prevalence of infections moderate or severe above 20% after deworming activities have been reported previously in Loreto Department although deworming coverage was below 75% [20]. Deworming reduces morbidity by reducing the individual worm burden, but it must be accompanied by education on health and hygiene to reduce transmission and reinfection by encouraging healthy behaviors as well as by provision of adequate sanitation [13,58].

We found that the prevalence of anemia (32.25%) in the surveyed children aged 1–9 years, is considered as a public health problem of moderate significance, based on the thresholds established by WHO [38]. Although we did not find an association between anemia and STH infections in children 1–9 years old in our study, anemia is associated with poor cognitive and motor development in children [59]. We found a prevalence of 23.10% of *T*. *trichiura* infections in girls 1–9 years old, a finding that is important since there are studies indicating that pregnant women infected with *T*. *trichiura* and hookworms are at higher risk of anemia [13]. No reduction of anemia in girls might predict anemia in women of reproductive age and pregnant women. Heavy-intensity STH infections have been described as a cause of loss of iron in school-age children [13], but in our study, most of the infections were mild to moderate-intense, and deworming has been carried out in those communities. This might explain our finding of no association between anemia and STH in children, but additional analysis is needed to understand the contribution of STH infections to anemia in the studied population.

We did not find an association between BMI and STH in the studied population. STH infections, especially moderate to heavy-intense, have effects on nutrition. For example, hookworms and *T*. *trichiura* can cause blood loss and *A*. *lumbricoides* can cause malabsorption and nutrient losses [13]. However, these effects are influenced by other factors such as coinfections with other parasites, deworming, nutrition interventions, etc. In our study, approximately 5% of children 1–5 years old were wasted or severely wasted which in conjunction with anemia and STH infections, are factors that can affect child development in these rural populations living already in vulnerable conditions. Wasting indicates acute malnutrition and increases the risk of death in childhood from infectious diseases such as diarrhea, pneumonia, and measles [60]. It is noticed, that approximately 20% of children aged 1–5 years in the studied population were at possible risk of being overweight or overweight and 18% aged 6–9 years, were overweight or obese. Childhood overweight and obesity are associated with a higher probability of overweight and obesity in adulthood, which can lead to various non-communicable diseases, such as diabetes and cardiovascular diseases [60]. Our findings are consistent with reports showing that while child malnutrition falls but still affects the poorest in Latin America and the Caribbean, overweight among children increases [60].

The co-occurrence of TF and STH was found in children aged 1–9 years in 13 out of the 20 clusters in the evaluation district. Children aged 3–8 years had an almost seven-fold increased odds of TF alone and four-fold of co-occurrence of TF and STH compared to children aged 1–2 years, while there was no difference in the odds for children aged nine years. Similar findings for trachoma have been described in other studies [61,62], and it could reflect decreased exposure to re-infection as children reach school age for both diseases [63,64]. The latter can be supported by the fact that the enrollment rate for initial education in children aged 3–5 years in Loreto Department is 78.20% and for basic education in children aged 6–12 years is higher than 90% [65].

The strong association between unclean faces and TF suggests that eye secretion, nasal discharge, and/or flies are important sources of transmission and support the need to implement actions to improve facial cleanliness in the evaluation district. These findings are consistent with the results of other studies [66–71], and support the importance of evaluating the clean face status of children in areas where trachoma is suspected, as it provides data on the route of transmission.

Children with unclean faces had also an increased odds of co-occurrence of TF and STH. Although most children were found to wash their hands before eating and after defecating, self-reported questions related to these behaviors might not be the best way to evaluate the STH-risk factors related to personal hygiene. Signs of unclean faces were observed by the interviewers during the fieldwork, while questions related to hygiene behaviors related to STH were asked either to the parents or guardians when children were unable to reply or directly to children. It could be possible that participants replied affirmatively when asked about personal hygiene behaviors to avoid the shame of a negative answer. Hence, unclean faces might be a proxy indicator of personal hygiene that is a factor related to both diseases.

Children that lived in households where the main source of water for washing faces of the household members was rainwater collection had an increased odds of co-occurrence of TF and STH. This may be related to the fecal contamination of water stored in households for consumption after collecting it from the rain or other sources as reported in other studies, in which fecal contamination of drinking water is widespread, particularly in rural areas and in low-income countries [72,73]. Although drinking contaminated water is not considered a typical route of STH infection, some studies have found helminth ova in samples drawn from the water storage container, suggesting the possibility of contamination during transport, storage, or usage of the water [73–76]. Contaminated water used for washing faces should not have an effect on trachoma, but it is an indicator of the availability of water for washing faces. We did not find an interaction between clean faces and the source of water for washing faces.

We found that being female was associated with a reduced odds of TF alone and of the co-occurrence of TF and STH in children aged 1 to 9 years. While many studies have found that the risk of active trachoma is higher in females [77–79], not all studies have found a female excess [62,67,80,81]. Women and children in rural communities of Peru without access to safe water are responsible for carrying water from several sources to the households [82]. According to official statistics in Peru, adolescents and young women dedicated 1.35 and 1.28 hours daily, respectively, in 2010 to carrying water from several sources to be used and stored in the households. Adolescents and young men dedicated 1.22 and 1.19 hours daily, respectively. We found that 26.61% of children aged 1–9 years lived in households where members wash their faces at the source of water which perhaps contributes to the decreased odds of TF in girls. Also, this finding might be related to some other behaviors, for example, boys sleeping together which could increase their odds of TF. It has been reported in some studies that trachoma prevalence increased in children with the number of people sleeping in the same room [83]. The reduced odds for the co-occurrence of TF and STH in females may be a proxy indicator of some personal hygiene behaviors that might protect them from STH. We did not find differences between the percentages of deworming by sex. This finding of reduced risk of TF and co-occurrence of TF and STH in girls should be studied in populations from the Amazon basin to understand better how this can be used for the implementation of interventions in these specific populations.

We found that children aged 1–9 years that were dewormed in the last six months had reduced odds of STH, as expected, but with increased odds of TF. The increased odds of TF in children dewormed is likely because both diseases overlap in the rural and poor communities of the Loreto Department, as previously mentioned, and these children would have had both diseases if they had not been dewormed.

There are some limitations in our study. The cross-sectional nature of the study design does not permit inferences of causality. While we can only conclude the risk factors are associated with the diseases of interest, there is some biological plausibility to support a causal relationship for some factors, such as drinking contaminated water and STH. Response bias may occur as some variables were collected based on self-reported information. Some findings might be influenced by this bias, such as hygiene behaviors related to STH like washing hands before eating and after defecating. There is a limitation in the estimation of the TT prevalence because the sample size was calculated to have good power to estimate the TF prevalence but is underpowered to reliably estimate the prevalence of TT, which is rare. However, data on TT gives us information useful for establishing interventions that include searching for TT cases in the area. Finally, we did not seek confirmation of all TF cases using images, as has been done in other work. The use of GTMP methods and standardization should help guarantee the reliability of grading TF, but over or under-calling is always a potential issue.

Findings in our study support the need to implement interventions of the SAFE strategy in the new trachoma endemic district [6], where some of the hygiene interventions should be structured around both trachoma and STH prevention. Based on the WHO’s recommendations [28], a round of mass drug administration using azithromycin should be carried out in endemic districts in all populations reaching 80% or higher coverage. An impact survey should be carried out two years later to evaluate the prevalence of TF in children.

Mass deworming should be sustained to keep the intensity of infection of STH as low as possible to reduce morbidity in children. Deworming should be expanded into other at-risk groups such as women of childbearing age, adolescent women, and pregnant women after the first trimester, with coverage reaching 75% or higher [13,84].

Access to safe water, sanitation, and hygiene education needs to be increased and sustained in rural communities of the Loreto Department to eliminate trachoma and STH. This should include the active involvement of local leaders and intersectoral actors to plan and adapt the interventions to the geographical, environmental, cultural, and socioeconomic characteristics of the population.

Trachoma needs to be mapped in other geographical municipalities of the Loreto Department to determine the extension of the affected communities, as well as in other departments of Peru where trachoma has been reported in previous studies [9]. Trachoma surveys should include the collection of data for conjunctival *C*. *trachomatis* infection and exposure to *C*. *trachomatis* infection to obtain complementary information to better characterize the epidemiology of the disease in the studied populations. Searching for TT cases is needed in Loreto Department to refer them to evaluation and eventually surgery to reduce progression to corneal opacity and eventually to blindness. Integration of trachoma actions with visual health initiatives in Peru may increase the capacity in the country to tackle the problem and to increase access to health services in communities living in remote and rural areas of the Amazon basin. Interprogrammatic actions are one of the recommended approaches in the neglected tropical diseases road map 2021–2030 of WHO [85] as well as in the PAHO’s initiative to eliminate communicable diseases by 2030 [86].

Neglected tropical diseases, in this case, trachoma and STH, their risk factors (lack of water and sanitation, difficult access to health services, etc.), and related morbidity (anemia, malnutrition, etc.), plus other health problems (overweight and obesity), overlap in the studied population. The Amazon basin is a geographical region of 7.8 million square kilometers with approximately 34 million people, 826 indigenous groups, and 200 more living in voluntary isolation [87,88]. Eight South American countries—Bolivia (Plurinational State of), Brazil, Colombia, Ecuador, Guyana, Peru, Suriname, and Venezuela (Bolivarian Republic of)—and French Guiana have populations living in the Amazon basin. This is a region that faces multiple challenges, where multilanguage, multicultural, and diverse populations still live in vulnerable conditions exacerbating their health problems and undermining their capacities to overcome poverty. Eliminating neglected tropical diseases in Peru and other countries in the Amazon basin demands a joint governmental response involving community leaders, social organizations, and public and private partners to leave no one behind. This should include guaranteeing the inclusion of interventions and specific budget lines in the national and subnational health and public health plans and mainstreaming of actions in the health system.

This study demonstrated that integrated approaches can be implemented, in this case, to compile data and information to contribute to designing, adapting, implementing, monitoring, and evaluating tailored integrated actions to eliminate neglected tropical diseases in hard-to-reach populations. Integrated approaches can help to overcome geographical and operational challenges that make it highly expensive to reach communities in areas such as the Amazon basin.

## Supporting information

S1 STROBE Checklist Checklist of items that should be included in reports of *cross-sectional studies*. (DOC)
