# Supplementary material for: Associated factors of the co-occurrence of trachoma and soil-transmitted helminthiases in children 1 to 9 years old in rural communities of the Amazon basin in Loreto Department, Peru: Results from a population-based survey
Source: PLoS Negl Trop Dis. 2022 Jul 25;16(7):e0010532. doi: 10.1371/journal.pntd.0010532 (PMC9312473; doi:10.1371/journal.pntd.0010532)
Supplement: S1 STROBE Checklist — (DOC) [file pntd.0010532.s001.doc]

STROBE Statement—Checklist of items that should be included in reports of ***cross-sectional studies***

|  | Item No | Recommendation |
| --- | --- | --- |
| **Title and abstract** | 1 | (*a*) Indicate the study’s design with a commonly used term in the title or the abstract  *It is indicated in the title as a population-based survey* |
|  |  | (*b*) Provide in the abstract an informative and balanced summary of what was done and what was found  *The abstract summarizes methods and findings* |
| Introduction | | |
| Background/rationale | 2 | Explain the scientific background and rationale for the investigation being reported  *Background and rational are described in the introduction, paragraphs 1-4* |
| Objectives | 3 | State specific objectives, including any prespecified hypotheses  *Objectives are described in introduction, paragraph 5* |
| Methods | | |
| Study design | 4 | Present key elements of study design early in the paper  *Study design is described in methods section, paragraph 4* |
| Setting | 5 | Describe the setting, locations, and relevant dates, including periods of recruitment, exposure, follow-up, and data collection  *Settings, locations, and period of implementation of the study are described in paragraphs 2 and 3 of the methods section*. |
| Participants | 6 | (*a*) Give the eligibility criteria, and the sources and methods of selection of participants  *Selection of participants is described in methods section, paragraphs 6, 7, and 8. Eligibility criteria are described in methods, paragraph 12, 13, 14 of methods section.* |
| Variables | 7 | Clearly define all outcomes, exposures, predictors, potential confounders, and effect modifiers. Give diagnostic criteria, if applicable  *Outcomes for clinical signs of trachoma, for STH parasitological indicators, and for risk factors for both diseases are defined in methods section, paragraphs 13 and 14* |
| Data sources/ measurement | 8* | For each variable of interest, give sources of data and details of methods of assessment (measurement). Describe comparability of assessment methods if there is more than one group  *Detail methods of measurement for the outcomes for trachoma and STH are described in methods section, paragraphs 10 to 14* |
| Bias | 9 | Describe any efforts to address potential sources of bias  *This was addressed in methods section, under survey teams – paragraphs 9 and 10* |
| Study size | 10 | Explain how the study size was arrived at  *This is described in methods section, under study design and sample size – paragraph 4* |
| Quantitative variables | 11 | Explain how quantitative variables were handled in the analyses. If applicable, describe which groupings were chosen and why  *Analysis is described under methods section, data collection, management and analysis, paragraphs 15-18* |
| Statistical methods | 12 | (*a*) Describe all statistical methods, including those used to control for confounding  *Described under methods section, data collection, management and analysis, paragraphs 15-18* |
|  |  | (*b*) Describe any methods used to examine subgroups and interactions  *Described under methods section, data collection, management and analysis, paragraphs 15-18* |
|  |  | (*c*) Explain how missing data were addressed  *This is described under results, paragraph 1, and then in the table notes of tables 5-7 and 8* |
|  |  | (*d*) If applicable, describe analytical methods taking account of sampling strategy  *This is described in methods section*, *data collection, management and analysis, paragraphs 15-18* |
|  |  | (*e*) Describe any sensitivity analyses  *(Not applicable)* |
| Results | | |
| Participants | 13* | (a) Report numbers of individuals at each stage of study—eg numbers potentially eligible, examined for eligibility, confirmed eligible, included in the study, completing follow-up, and analysed  *This is described in results section, population surveyed – paragraph 1* |
|  |  | (b) Give reasons for non-participation at each stage  *This is described in results section, population surveyed – paragraph 1* |
|  |  | (c) Consider use of a flow diagram  (*Not applicable)* |
| Descriptive data | 14* | (a) Give characteristics of study participants (eg demographic, clinical, social) and information on exposures and potential confounders  *This is described under results section, paragraph 1, table 2* |
|  |  | (b) Indicate number of participants with missing data for each variable of interest  *This is described under results, paragraph 1, and then in the table notes of tables 5-7 and 8* |
| Outcome data | 15* | Report numbers of outcome events or summary measures  *This is described under results, paragraph 2-7, and tables 3-7* |
| Main results | 16 | (*a*) Give unadjusted estimates and, if applicable, confounder-adjusted estimates and their precision (eg, 95% confidence interval). Make clear which confounders were adjusted for and why they were included  *This is described under results, paragraph 2-10, and tables 3-10* |
|  |  | (*b*) Report category boundaries when continuous variables were categorized  *(Not applicable)* |
|  |  | (*c*) If relevant, consider translating estimates of relative risk into absolute risk for a meaningful time period  *(Not applicable)* |
| Other analyses | 17 | Report other analyses done—eg analyses of subgroups and interactions, and sensitivity analyses  *(Not applicable)* |
| Discussion | | |
| Key results | 18 | Summarise key results with reference to study objectives  *This is described under discussion section, paragraphs 2-15* |
| Limitations | 19 | Discuss limitations of the study, taking into account sources of potential bias or imprecision. Discuss both direction and magnitude of any potential bias  *This is described under discussion section, paragraphs 16* |
| Interpretation | 20 | Give a cautious overall interpretation of results considering objectives, limitations, multiplicity of analyses, results from similar studies, and other relevant evidence  *This is described under discussion section, paragraphs 17-21* |
| Generalisability | 21 | Discuss the generalisability (external validity) of the study results  *This is described under discussion section, paragraphs 19* |
| Other information | | |
| Funding | 22 | Give the source of funding and the role of the funders for the present study and, if applicable, for the original study on which the present article is based  *The following sections are included right after the discussion describing authors contributions, funding, and competing interest* |

*Give information separately for exposed and unexposed groups.

**Note:** An Explanation and Elaboration article discusses each checklist item and gives methodological background and published examples of transparent reporting. The STROBE checklist is best used in conjunction with this article (freely available on the Web sites of PLoS Medicine at http://www.plosmedicine.org/, Annals of Internal Medicine at http://www.annals.org/, and Epidemiology at http://www.epidem.com/). Information on the STROBE Initiative is available at www.strobe-statement.org.
